# Analysis of outcomes and prognostic factor in acute type A aortic dissection complicated with preoperative shock: A single-center study

**DOI:** 10.1371/journal.pone.0302669

**Published:** 2024-04-30

**Authors:** Chun-Yu Lin, Ming-Chang Kao, Hsin-Fu Lee, Meng-Yu Wu, Chi-Nan Tseng

**Affiliations:** 1 Department of Medicine, College of Medicine, Chang Gung University, Taoyuan City, Taiwan; 2 Department of Cardiothoracic and Vascular Surgery, New Taipei Municipal TuCheng Hospital, New Taipei City, Taiwan; 3 Department of Anesthesiology, New Taipei Municipal TuCheng Hospital, New Taipei City, Taiwan; 4 Department of Cardiology, New Taipei Municipal TuCheng Hospital, New Taipei City, Taiwan; 5 Department of Cardiothoracic and Vascular Surgery, Chang Gung Memorial Hospital, Linkou Medical Center, Taoyuan City, Taiwan; Scuola Superiore Sant’Anna, ITALY

## Abstract

**Background:**

Acute type A aortic dissection (ATAAD) is a critical cardiovascular emergency that requires prompt surgical intervention for preserving life, particularly in patients with critical preoperative status. This retrospective study aimed to investigate the clinical features, early and late outcomes, and prognostic factors in patients undergoing aortic repair surgery for ATAAD complicated with preoperative shock.

**Methods:**

Between April 2007 and July 2020, 694 consecutive patients underwent emergency ATAAD repair at our institution, including 162 (23.3%) presenting with preoperative shock (systolic blood pressure <90 mm Hg), who were classified into the survivor (n = 125) and non-survivor (n = 37) groups according to whether they survived to hospital discharge. The clinical demographics, surgical information, and postoperative complications were compared. Five-year survival and freedom from reoperation rates of survivors were analyzed using the Kaplan–Meier actuarial method. Multivariate logistic regression analysis was used to identify independent risk factors for in-hospital mortality.

**Results:**

The in-hospital surgical mortality rate in patients with ATAAD and shock was 22.8%. The non-survivor group showed higher rates of preoperative cardiopulmonary resuscitation, acute myocardial infarction, and cerebral infarction, and was associated with longer cardiopulmonary bypass time, higher rates of total arch replacement and intraoperative extracorporeal membrane oxygenation implementation. The non-survivor group had higher blood transfusion volumes and rates of malperfusion-related complications. Multivariate analysis revealed that preoperative cardiopulmonary resuscitation, prolonged cardiopulmonary bypass time, and total arch replacement were risk factors for in-hospital mortality. For patients who survived to discharge, the 5-year cumulative survival and freedom from aortic reoperation rates were 75.6% (95% confidence interval, 67.6%–83.6%) and 82.6% (95% confidence interval, 74.2%–91.1%), respectively.

**Conclusions:**

Preoperative shock in ATAAD is associated with a high risk of in-hospital mortality, particularly in patients who undergo cardiopulmonary resuscitation and complex aortic repair procedures with extended cardiopulmonary bypass. However, late outcomes are acceptable for patients who were stabilized through surgical treatment and survived to discharge.

## Introduction

Acute type A aortic dissection (ATAAD) is a critical cardiovascular disease associated with high mortality and perioperative complication rates [1,2]. Prompt surgical intervention is essential to save lives, particularly in patients presenting with hemodynamic instability, rupture of the dissected aorta, or malperfusion of vital organs. It is also challenging for cardiothoracic surgeons because of the individual patient’s complexity of aorta anatomy and the rapid progression of disease. Despite improvements in management algorithms, surgical strategies, and cardiopulmonary bypass (CPB) techniques in the last few decades, preoperative hypotensive shock remains an important prognostic factor for ATAAD treatment [3]. The reported incidence of preoperative shock in the ATAAD population is 16–57% according to different international aortic dissection data registries [3–6]. It is associated with adverse clinical outcomes, including reduced surgical survival rates and increased risks of malperfusion-related complications such as cerebral injury and myocardial ischemia in patients undergoing ATAAD repair surgery [7]. Nevertheless, a detailed comparison of the clinical features and surgical outcomes between survivors and non-survivors in this high-risk population has been underreported in previous literatures. In this study, we performed a retrospective analysis using a database from an individual aortic intervention center to investigate the clinical demographics, surgical information, early and late outcomes, and prognostic factors of patients who underwent aortic repair surgery for ATAAD complicated with preoperative hypotensive shock.

## Materials and methods

### Patient enrollment and preoperative management

**Fig 1:**
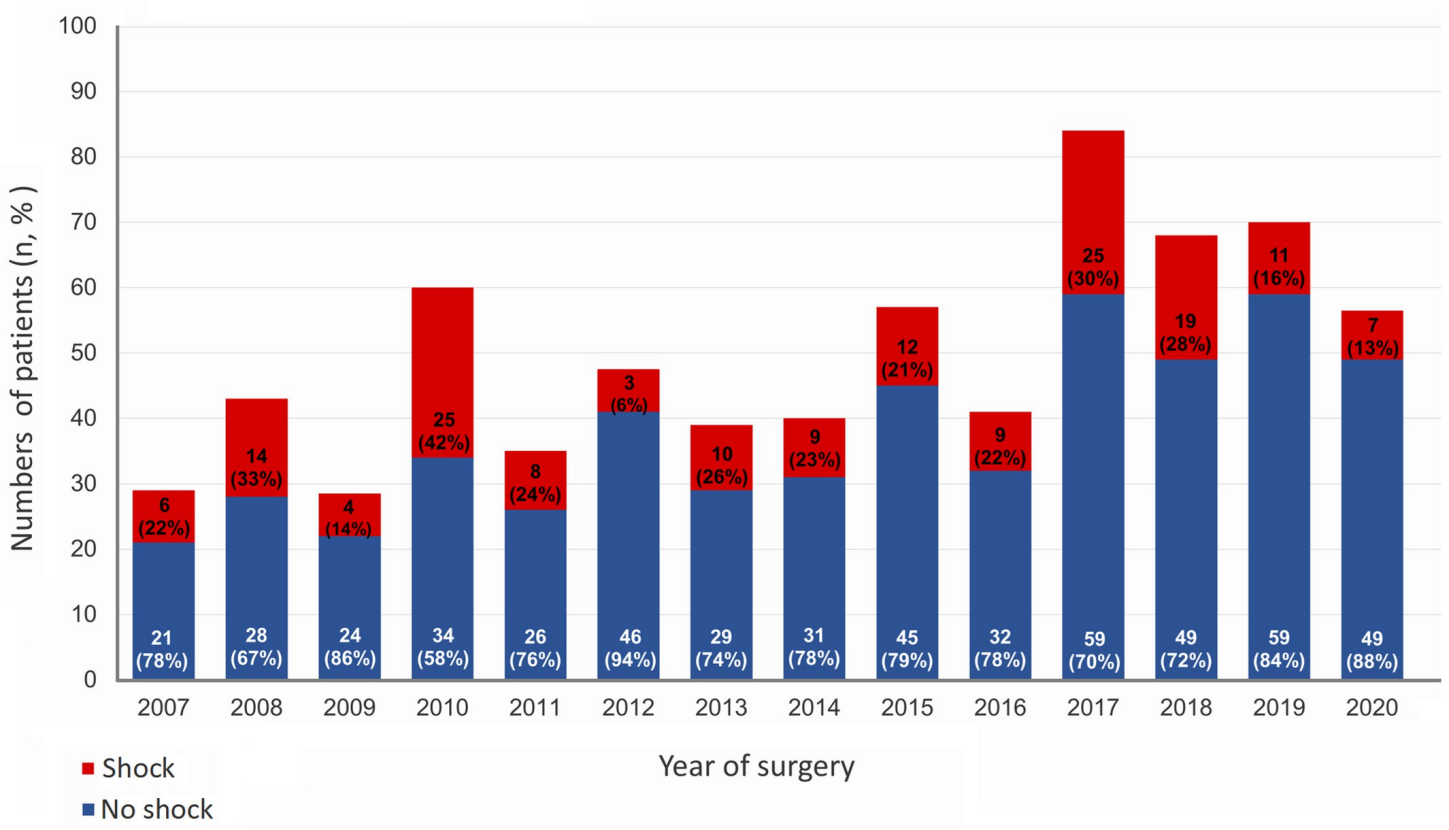
Annual cases in the overall cohort, survivor group, and non-survivor group during the study period.

The study protocol was approved by the Institutional Review Board of Chang Gung Medical Foundation (registration number 202301843B0). All data were accessed and analyzed using the institutional database of aortic dissection. The data were accessed for research purposes on December 18, 2023. The need for informed consent was waived due to the retrospective nature of the study, and all patients’ data were anonymized before being accessed by researchers. A total of 694 consecutive patient records undergoing emergency aortic repair surgery for ATAAD at our institution were collected between April 2007 and July 2020. After excluding those hemodynamically stable before surgery, this study investigated 162 patients classified with preoperative hypotensive shock (systolic blood pressure <90 mmHg) according to the vital sign data recorded upon emergency department admission. The annual cases of the overall cohort, shock, and non-shock patients during the study period are shown in Fig 1. All patients were diagnosed using helical computed tomography and transferred to the operating room for emergency aortic repair surgery within 60 min after the initial diagnosis. The 162 investigated patients were dichotomized into the survivor (n = 125) and non-survivor (n = 37) groups according to whether they survived to hospital discharge. For preoperative hemodynamic support, medical resuscitation was applied first, including intravenous fluid supplementation and/or inotropic infusion, according to the established guidelines for advanced cardiovascular life support (ACLS) developed by the American Heart Association during the study period [8–10]. Cardiopulmonary resuscitation (CPR) and/or surgical rescue managements were implemented promptly if patients presented with refractory hemodynamic instability or cardiac arrest based on the standardized protocols of our institute [11,12]. Patients underwent standard external CPR, which was performed by physicians with ACLS certification. Surgical resuscitation procedures, including emergent subxiphoid pericardiotomy and emergent cardiopulmonary bypass, were implemented according to the individual patient’s circumstance. In general, emergent subxiphoid pericardiotomy was performed if cardiac tamponade was confirmed based on preoperative imaging studies and on-site echocardiography; emergent cardiopulmonary bypass was performed if aortic rupture was suspected or if patients presented with refractory cardiac arrest without return of spontaneous circulation after CPR and medical resuscitation. European system for cardiac operative risk evaluation score (EuroSCORE) II, Leontyev score, and Rampoldi score were used for evaluate the surgical risk [13–15].

### Aortic repair procedures and postoperative treatment

The aortic repair procedures for ATAAD were standardized at this institute, as discussed in previous studies [16,17]. For patients who presented with unstable conditions preoperatively, including shock, CPR, and vital organ malperfusion, femoral artery cannulation was commonly adapted first to establish prompt CPB to stabilize the patient’s hemodynamic status. Additional right axillary artery cannulation was used with combining the femoral arterial access, which generated double artery cannulation with antegrade cerebral perfusion strategy if the patient’s hemodynamics were relatively stabilized under medication and CPB. In contrast, isolated femoral artery cannulation combining superior vena cava cannulation with retrograde cerebral perfusion was preferred for patients with persistent critical hemodynamics refractory to medical and surgical resuscitation. Full sternotomy was routinely performed in all patients. Following the cannulation of the right atrium or vena cava, CPB with systemic hypothermia was performed. The tubular ascending aorta (AsAo) was completely resected and replaced with Dacron prosthetic grafts. Proximal anastomosis is usually performed first, followed by open distal anastomosis under deep hypothermic circulatory arrest (18–22°C). In general, the dissected aorta was replaced with Dacron prosthetic grafts according to the location of the entry tears and preoperative presentation. Concomitant aortic root and arch replacement were performed using a composite Valsava graft and branched Dacron graft, respectively, according to the location of the entry tear and preoperative clinical presentation, if feasible. All graft-aorta anastomoses were reinforced with Teflon strips and surgical sealants. During circulatory arrest, the femoral arterial flow was temporarily suspended, and selective antegrade cerebral perfusion through the right axillary artery or retrograde cerebral perfusion through the superior vena cava was implemented depending on the vascular access of CPB. After surgical repair of ATAAD, all patients were transferred to a specialized cardiovascular intensive care unit for further treatment and close observation of postoperative complications, including bleeding tendencies, arrhythmia, myocardial failure, organ malperfusion, and hemodynamic instability.

### Statistical analyses

Statistical analyses were performed using SPSS for Windows (version 26.0; IBM Corp., Armonk, NY, USA). Data are presented as means ± standard deviation for continuous variables and numbers (n) and percentages (%) for categorical variables, respectively. To compare the intergroup disparities between the survivor and non-survivor groups, we used an independent t-test for continuous variables and the chi-square test for categorical variables, respectively. The preoperative and surgical variables were first tested using univariate logistic regression analysis. The significant variables in the univariate logistic regression analysis were further analyzed via the multivariate logistic regression analysis to identify the independent risk factors for in-hospital mortality. The Kaplan–Meier method was used to estimate the 5-year cumulative survival and freedom from aortic reoperation rates of the survivors. For all analyses, the statistical significance was set at *P*<0.05.

## Results

### Patient demographics

Table 1 lists the preoperative demographics, which showed no significant differences in age, sex, and chronic comorbidities. The mean age was 61.9±13.8 years, and 36.4% of the patients were females. The non-survivor group revealed a more critical preoperative condition, including lower systolic blood pressure (73.5±11.5 mmHg versus 68.3±14.7 mmHg; *P* = 0.027), higher rates of CPR (8.0% versus 35.1%; *P*<0.001), cerebral infarction (3.2% versus 13.5%; *P* = 0.016), myocardial infarction (0.8% versus 8.1%; *P* = 0.012), a higher EuroSCORE II estimated in-hospital mortality rate (22.1±3.9% versus 54.5±5.6%; *P*<0.001), higher Leontyev score (5.3±1.6 versus 9.6±1.5; *P*<0.001) and Rampoldi score (1.8±0.4 versus 3.8±0.5; *P*<0.001). Chest or back pain was the most common clinical presentation in both groups, followed by hemopericardium/cardiac tamponade, organ malperfusion, and severe aortic regurgitation.

**Table 1:** Preoperative characteristics according to patient group.

Parameters	Total	Survivors	Non-survivors	*P* value
	n = 162	n = 125	n = 37	
Clinical demographics				
Age (years)	61.9±13.8	62.1±13.3	61.1±15.6	0.697
Sex (female, n,%)	59, 36.4	50, 40.0	9, 24.3	0.082
Hypertension (n,%)	111, 68.5	87, 69.6	24, 64.9	0.586
Diabetes mellitus (n,%)	10, 6.2	8, 6.4	2, 5.4	0.825
Creatinine (mg/dL)	1.5±1.3	1.5±1.5	1.4±0.5	0.531
eGFR (mL/min/1.73 m_2_)	61.2±29.7	61.4±31.2	60.6±24.6	0.882
End-stage renal disease (n,%)	2, 1.2	2, 1.6	0	0.439
Preoperative condition				
Systolic blood pressure (mmHg)	72.3±12.4	73.5±11.5	68.3±14.7	0.027
Systolic blood pressure <60 mmHg (n,%)	33, 20.4	22, 17.6	11, 29.7	0.108
Cardiopulmonary resuscitation (n,%)	23, 14.2	10, 8.0	13, 35.1	<0.001
Ventilator support (n,%)	21, 13.0	13, 10.4	8, 21.6	0.074
Repeat surgery (n,%)	7, 4.3	4, 3.2	3, 8.1	0.197
Time from ED to OR (h)	5.0±2.9	5.1±3.0	4.9±2.5	0.688
EuroSCORE II (%)	29.5±14.3	22.1±3.9	54.5±5.6	<0.001
Leontyev score	6.3±2.4	5.3±1.6	9.6±1.5	<0.001
Rampoldi score	2.3±1.0	1.8±0.4	3.8±0.5	<0.001
Clinical presentation				
Chest/back pain (n,%)	110, 67.9	83, 66.4	27, 73.0	0.452
Hemopericardium (n,%)	84, 51.9	67, 53.6	17, 45.9	0.413
Cardiac tamponade (n,%)	56, 34.6	47, 37.6	9, 24.3	0.136
Aortic regurgitation > moderate (n,%)	26, 16.0	19, 15.2	7, 18.9	0.588
Malperfusion^a^ (n,%)	30, 18.5	21, 16.8	9, 24.3	0.301
Cerebral infarction (n,%)	9, 5.6	4, 3.2	5, 13.5	0.016
Myocardial infarction (n,%)	4, 2.5	1, 0.8	3, 8.1	0.012

^a^Occurrence of preoperative cerebral infarction, paraplegia, myocardial infarction, mesenteric ischemia, and limb ischemia.

ED, emergency department; eGFR, estimated glomerular filtration rate; EuroSCORE II, European system for cardiac operative risk evaluation score II; OR, operating room.

### Surgical information

Table 2 provides detailed information on surgical variables. The vascular access of cannulation and cerebral perfusion strategies did not differ significantly between the two groups. However, the non-survivor group exhibited a higher rate of total arch replacement (4.8% versus 16.2%; *P* = 0.020), as well as longer CPB (249.5±70.8 min versus 316.7±117.3 min; *P*<0.001) and aortic clamping (161.4±51.4 min versus 186.5±79.1 min; *P*<0.001) times. A higher rate of intraoperative myocardial failure with extracorporeal membrane oxygenation support (2.4% versus 10.8%; *P* = 0.027) was observed in the non-survivor group.

**Table 2:** Surgical information according to patient group.

Parameters	Total	Survivors	Non-survivors	*P* value
	n = 162	n = 125	n = 37	
Surgical resuscitation procedures (n,%)	49, 30.2	35, 28.0	14, 37.8	0.252
Emergent subxiphoid pericardiotomy(n,%)	33, 20.4	24, 19.2	9, 24.3	0.497
Emergent cardiopulmonary bypass (n,%)	25, 15.4	16, 12.8	9, 24.3	0.088
Femoral artery cannulation (n,%)	156, 96.3	119, 95.2	37, 100	0.174
Axillary artery cannulation (n,%)	118, 72.8	94, 75.2	24, 64.9	0.214
Aortic repair procedures				
Isolated AsAo replacement (n,%)	118, 72.8	93, 74.4	24, 64.9	0.255
Root replacement (n,%)	18, 11.1	12, 9.6	6, 16.2	0.261
Arch replacement (n,%)	31, 19.1	22, 17.6	9, 24.3	0.361
Partial arch (n,%)	19, 11.7	16, 12.8	3, 8.1	0.436
Total arch (n,%)	12, 7.4	6, 4.8	6, 16.2	0.020
Frozen elephant trunk (n,%)	10, 6.2	6, 4.8	4, 10.8	0.182
Resection of entry tear (n,%)	115, 71	88, 70.4	27, 73.0	0.762
CABG (n,%)	7, 4.3	5, 4.0	2, 5.4	0.197
Cardiopulmonary bypass time (min)	264.8±87.9	249.5±70.8	316.7±117.3	<0.001
Aortic clamping time (min)	167.1±59.6	161.4±51.4	186.5±79.1	0.024
Circulatory arrest time (min)	48.6±20.2	47.0±18.8	53.9±23.7	0.066
Hypothermia temperature (°C)	20.2±2.6	20.4±2.7	19.5±1.6	0.051
Antegrade cerebral perfusion (n,%)	121, 74.7	97, 77.6	24, 64.9	0.118
Retrograde cerebral perfusion (n,%)	41, 25.3	28, 22.4	13, 35.1	0.118
Delayed sternal closure (n,%)	29, 17.9	24, 19.2	5, 13.5	0.428
Extracorporeal membrane oxygenation (n,%)	7, 4.3	3, 2.4	4, 10.8	0.027

AsAo, ascending aorta; CABG, Coronary artery bypass grafting.

### Postoperative complications

Table 3 shows the postoperative mortality and morbidity. The overall in-hospital mortality rate was 22.8%. Myocardial failure was the most common cause of mortality (40.5%), followed by bleeding (27.0%), brain stem failure (21.6%), and sepsis (10.8%). The blood transfusion volumes within 24 h after surgery were higher in the non-survivor group. The non-survivor group also had higher rates of malperfusion-related complications, including brain infarction (12.8% versus 32.4%; *P* = 0.006) and limb ischemia (1.6% versus 10.8%; *P* = 0.009) than the survivor group.

**Table 3:** Postoperative mortality and morbidity according to patient group.

Parameters	Total	Survivors	Non-survivors	*P* value
	n = 162	n = 125	n = 37	
Cause of in-hospital mortality				
Bleeding (n,%)	—	—	10, 27.0	N/A
Myocardial failure (n,%)	—	—	15, 40.5	N/A
Brain stem failure (n,%)	—	—	8, 21.6	N/A
Sepsis (n,%)	—	—	4, 10.8	N/A
Transfusion within 24 h after surgery				
RBC^a^ (units)	10.0±9.8	9.0±7.3	13.5±15.3	0.016
Plasma^b^ (units)	8.8±8.1	7.9±6.5	11.7±11.8	0.012
Platelet (units)	20.4±15.7	19.3±13.5	24.3±21.3	0.087
Reexploration for bleeding (n,%)	27, 16.7	20, 16.0	7, 18.9	0.676
Delirium (n,%)	35, 21.6	27,21.6	8, 21.6	0.998
Seizure (n,%)	13, 8.0	11, 8.8	2, 5.4	0.504
Brain stroke (n,%)	30, 18.5	18, 14.4	12, 32.4	0.013
Infarction (n,%)	28, 17.3	16,12.8	12, 32.4	0.006
Hemorrhage (n,%)	5, 3.1	4, 3.2	1, 2.7	0.878
Renal failure (n,%)	17, 10.5	13, 10.4	4, 10.8	0.943
Mesenteric ischemia (n,%)	6, 3.7	4, 3.2	2, 5.4	0.533
Limb ischemia (n,%)	6, 3.7	2, 1.6	4, 10.8	0.009
Extubation time (h)	101.7±171.1	73.1±83.9	198.4±307.0	<0.001
Ventilator support > 72 h (n,%)	61, 37.7	47, 37.6	14, 37.8	0.979
ICU stay (days)	7.9±10.9	7.7±9.0	8.7±15.9	0.631
Hospital stay (days)	22.7±20.0	26.8±19.2	8.8±16.1	<0.001

^a^Red blood cell transfusion includes the amount of whole blood and packed red cell concentrate.

^b^Plasma transfusion includes the amount of fresh-frozen plasma and cryoprecipitate.

ICU, intensive care unit.

### Risk factors associated with in-hospital mortality

Table 4 shows the logistic regression analyses for patients with shock at risk of in-hospital mortality, including preoperative systolic blood pressure, CPR, myocardial infarction, cerebral infarction, total arch replacement, CPB time, aortic clamping time, and intraoperative extracorporeal membrane oxygenation implementation. Three significant risk factors were identified: preoperative CPR (odds ratio [OR], 5.60; 95% confidence interval [CI], 1.10–28.62; *P* = 0.038), total arch replacement (OR, 4.98; 95% CI, 1.21–20.55; *P* = 0.026), and CPB time (OR, 1.01; 95% CI, 1.00–1.02; *P* = 0.005).

**Table 4:** Logistic regression analyses for in-hospital mortality.

Parameters	β-coefficient	Standard error	Odds ratio, 95% CI	*P* value
Univariate logistic regression				
Systolic blood pressure (mmHg)	-0.032	0.015	0.97 (0.94–1.00)	0.029
Cardiopulmonary resuscitation	1.829	0.477	6.23 (2.45–15.86)	<0.001
Acute myocardial infarction	2.393	1.171	10.94 (1.10–108.56)	0.041
Cerebral infarction	1.553	0.700	4.73 (1.20–18.63)	0.026
Total arch replacement	1.345	0.612	3.84 (1.16–12.73)	0.028
Cardiopulmonary bypass time (min)	0.008	0.002	1.01 (1.00–1.01)	<0.001
Aortic clamping time (min)	0.007	0.003	1.01 (1.00–1.01)	0.028
Extracorporeal membrane oxygenation	1.595	0.789	4.93 (1.05–23.12)	0.043
Multivariate logistic regression				
Cardiopulmonary resuscitation	1.723	0.832	5.60 (1.10–28.62)	0.038
Total arch replacement	1.606	0.723	4.98 (1.21–20.55)	0.026
Cardiopulmonary bypass time (min)	0.013	0.005	1.01 (1.00–1.02)	0.005

### Cumulative 5-year survival and freedom from reoperation rates

**Fig 2:**
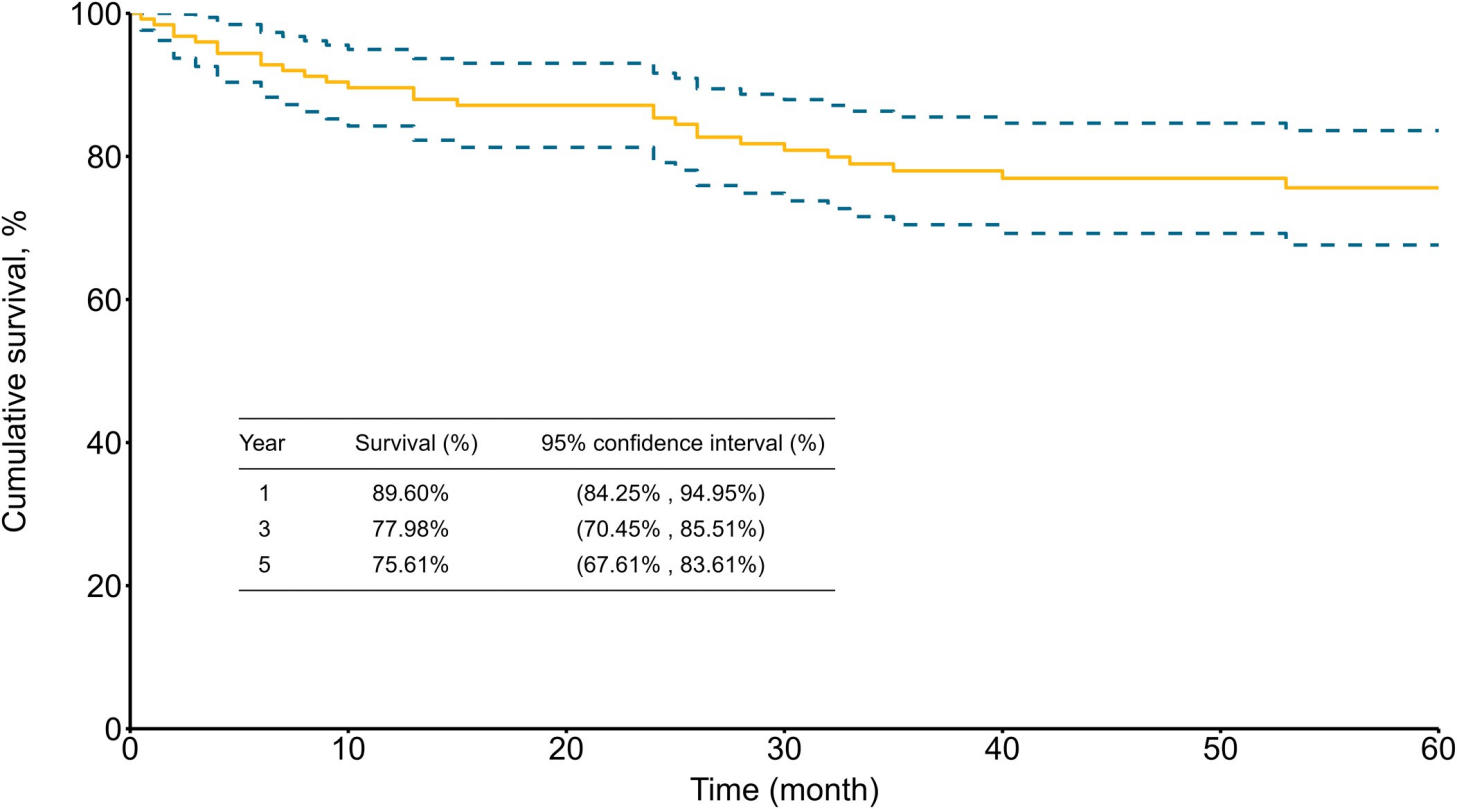
Five-year cumulative survival rates for 125 patients who survived to discharge.

**Fig 3:**
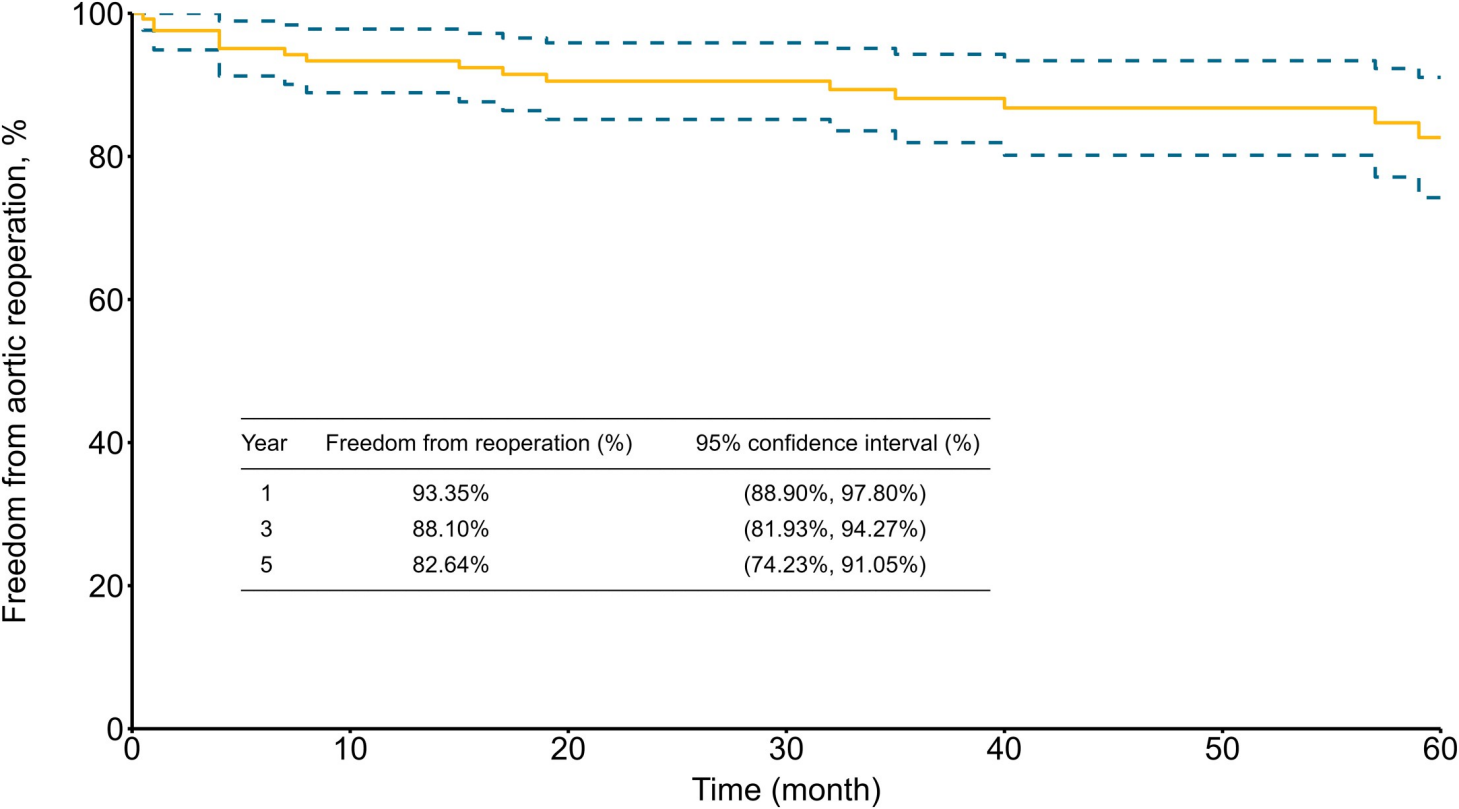
Five-year freedom from aortic reoperation rates for 125 patients who survived to discharge.

The average duration of follow-up was 4.8 ± 3.9 years (median, 4.2; range, 0.1–14.5 years). The cumulative survival rates for patients who survived to discharge were 89.60% (95% CI, 84.25%–94.95%), 77.98% (95% CI, 70.45%–85.51%), and 75.61% (95% CI, 67.61%–83.61%) at 1, 3, and 5 years, respectively (Fig 2). The freedom from aortic reoperation rates were 93.35% (95% CI, 88.90%–97.80%), 88.10% (95% CI, 81.93%–94.27%), and 82.64% (95% CI, 74.23%–91.05%) at 1, 3, and 5 years, respectively (Fig 3).

## Discussion

Presenting low blood pressure is reportedly a major risk factor for mortality and adverse events in the treatment of cardiovascular emergencies, including acute coronary syndromes, cardiogenic shock, acute heart failure, and acute aortic dissection [3,18,19]. However, comparisons of clinical features and surgical outcomes between the survivors and non-survivors among patients undergoing ATAAD repair complicated with preoperative shock have been underreported in previous studies. In this retrospective cohort study, we investigated 162 consecutive patients (125 survived and 37 non-survived to hospital discharge) who underwent emergency aortic repair for ATAAD during the study period. This study had several clinical implications. First, the incidence of preoperative hypotensive shock (162/694; 23.3%) was considerable in the ATAAD population and associated with a high risk of mortality and perioperative complications. Second, patients who underwent preoperative CPR, total arch replacement, and prolonged CPB time are at risk for in-hospital mortality in ATAAD with shock. Patients complicated by shock, particularly those with CPR, may adapt to conservative surgical strategies under comprehensive preoperative assessment to reduce the complexity of aortic repair procedure and subsequent complications, if feasible. Third, with timely surgical treatment to stabilize critical hemodynamics, patients who survived to discharge had acceptable late outcomes, including the 5-year survival and aortic reoperation rates.

The pathophysiology associated with shock complicated by ATAAD could be multifactorial, including malperfused vital organ systems, severe neurological dysfunction, acute heart failure, cardiac tamponade, and aortic rupture [20,21]. Furthermore, cardiac tamponade caused by hemorrhagic pericardial effusion leaking from the dissected AsAo is the leading cause of preoperative hemodynamic instability and death in patients with ATAAD before they present for emergency medical intervention [22,23]. It is also identified as a primary risk factor for perioperative mortality and morbidity [24,25]. In dissected AsAo, the persistent pressurized aortic false lumen could cause elastic microstructure injuries of the adventitia and aortic wall weakening, which could lead to severe bleeding or even frank rupture of AsAo into the pericardial space. Rapid accumulation of hemorrhagic pericardial fluid under pressure would compress the cardiac chambers, causing the blockage of systemic venous return to the right atrium and compromised cardiac output. Furthermore, this severe complication is also characterized by increased risks of organ malperfusion, neurologic deficits, and postoperative bleeding owing to the influence of unstable hemodynamics and related consumption coagulopathy.

The reported incidence of hemopericardium is approximately 24–29% in the general ATAAD population [26,27]. In contrast, 30–61% of patients in ATAAD with shock were complicated by periaortic hematoma or hemopericardium [3,7]. Similar outcomes were observed in this study. A total of 51.9% of patients were diagnosed with preoperative hemopericardium according to imaging studies, 34.6% of whom presented with cardiac tamponade. In previous studies reported from this institute investigating the general ATAAD population, the incidence rates of preoperative hemopericardium and cardiac tamponade were only approximately 28–33% and 11–13%, respectively [12,15,28,29]. At our institute, an aggressive surgical rescue strategy was adopted for this high-risk subgroup. A total of 30.2% of patients underwent surgical resuscitation procedures, including 20.4% of emergent subxiphoid pericardiotomy and 15.4% of emergent CPB, to temporarily stabilize the patient’s critical hemodynamics before implementing aortic repair surgeries. Furthermore, we found that the in-hospital mortality rates were 16.1% (9/56) and 26.4% (28/106) in patients with shock caused by cardiac tamponade and other etiologies, respectively. This result could be explained by that prompt and accurate management including medical resuscitation and surgical rescue procedures, would efficiently reverse the critical hemodynamics resulting from cardiac tamponade. Therefore, the treatment of ATAAD complicated by cardiac tamponade should be prioritized to achieve better survival. We suggest that an aggressive strategy of surgical rescue and early management decisions based on timely diagnostic studies are essential to stabilize patients with refractory hemodynamic instability and optimize surgical outcomes in this disastrous scenario. Otherwise, a large proportion of patients may not survive until surgery.

This study identified several risk factors associated with in-hospital mortality, including preoperative CPR for hemodynamic collapse. The reported incidence of preoperative CPR ranges from 3.4% to 6.8% in previous studies [12,30,31]. As a recognized preoperative risk factor for ATAAD, the early surgical mortality rate ranges from 43% to 95% [12,30,32], which is approximately three to five times higher than that of the general ATAAD population [1,2]. In the present study, 14.2% (23/162) of patients underwent CPR before ATAAD repair, and 56.5% (13/23) did not survive to discharge. We suggest several interpretations for this unfavorable outcome. First, manual external cardiac compression can generate only 20–30% of the normal cardiac output under standard CPR [33]. Furthermore, maintaining the quality of external cardiac massage during the emergency transportation of patients is challenging, prolonging the time of systemic hypoperfusion before surgical intervention. The performer’s fatigue could also reduce CPR efficiency. In this low-flow state, tissue hypoxia persists until effective spontaneous perfusion is restored, or extracorporeal circulation is initiated. However, an irreversible damage could have developed to the vital organs, particularly the heart and brain. Second, according to previous studies, if patients do not return to spontaneous circulation after utilizing immediate medical and surgical resuscitation, the mortality rate is high, which ranges 57–100% [12,30,34]. In this study, a total 14.2% of patients underwent CPR and 30.2% underwent surgical resuscitation procedures before surgery. Seven patients did not return to spontaneous circulation after resuscitation, and only one patient survived after surgery. According to our experience, most of these patients presented with extensive non-viable myocardial tissue and significant cardiac failure after sternotomy. Furthermore, the cardiac contractility of these patients generally showed limited improvement after completion of the aortic repair procedure. These patients usually died within 48 h after surgery. Except for surgical intervention, no further effective treatments are available for ATAAD post CPR, and decisions about the discontinuation of life-saving processes could be difficult after making huge efforts for emergency preoperative treatment and in consideration of the will of patients and their families [34]. However, we suggest that conservative surgical strategies may be reasonable for patients complicating with refractory cardiopulmonary failure without spontaneous circulation because an extremely unfavorable survival is expected. Furthermore, patients complicated by shock, particularly those with CPR, may adapt to conservative aortic repair strategies under comprehensive preoperative assessment to reduce the complexity of surgical procedure and CPB duration for optimal outcome, if feasible. Several operative risk scoring systems, including EuroSCORE II, Leontyev score, and Rampoldi score were used to evaluate the surgical risk for patients undergoing ATAAD repair in this study. The non-survivor group revealed significant higher scores in all of these risk-prediction models, which were consistent with the observed outcomes in the present study. Therefore, in addition to preoperative CPR, surgeons may also take into consideration the appropriate surgical strategies for patients exhibiting high operative risks based on the established prediction systems.

### Limitations

This study had several limitations. First, as a retrospective and non-randomized controlled study, potential bias may have existed that could influence the homogeneity of preoperative and operative variables between the investigated groups. Furthermore, the pathophysiology associated with shock complicated by ATAAD could be complex and multifactorial. The definite cause of preoperative shock for each individual patient might be difficult to clearly categorize. Second, the treatment protocols for ATAAD were based on the institutional consensus and established guidelines. However, the final decision was at the operating surgeon’s discretion, according to a comprehensive consideration of each individual patient’s clinical condition. Therefore, a conservative aortic repair strategy may be implemented in patients with critical conditions, including cardiopulmonary failure, sustained hypotension, CPR, and aortic rupture detected through imaging studies. Third, because this retrospective cohort study spanned a period of nearly 14 years, the technology of CPB, myocardial protection, and cerebral protection strategies for ATAAD surgery, as well as advanced cardiovascular life support management and intensive care protocols may have changed over time. Finally, despite the substantial early and late outcomes presented in this study, an extended follow-up study should be conducted in the future to further analyze the long-term outcomes in patients who undergo surgical treatment for ATAAD complicated by preoperative shock.

## Conclusions

The presence of hypotensive shock before ATAAD repair surgery is associated with a high risk of in-hospital mortality and postoperative complications, particularly in patients who undergo cardiopulmonary resuscitation, complex aortic repair procedures, and prolonged cardiopulmonary bypass time. However, once these high-risk patients are stabilized through surgery and survive to hospital discharge, the late outcomes, including survival and aortic reoperation rates during a 5-year follow-up, are acceptable.
